# Case Report: Multisystem Autoimmune and Overlapping GAD65-Antibody-Associated Neurological Disorders With Beneficial Effect of Epilepsy Surgery and Rituximab Treatment

**DOI:** 10.3389/fneur.2021.756668

**Published:** 2022-01-20

**Authors:** Petia Dimova, Krassimir Minkin

**Affiliations:** ^1^Epileptology Unit at Epilepsy Surgery Center, Department of Neurosurgery, St. Ivan Rilski University Hospital, Sofia, Bulgaria; ^2^Functional and Epilepsy Surgery Center, Department of Neurosurgery, St. Ivan Rilski University Hospital, Sofia, Bulgaria

**Keywords:** autoimmune, GAD, cerebellar, diabetes, epilepsy, insular, temporal, surgery

## Abstract

Glutamic acid decarboxylase (GAD) antibodies are associated with disabling conditions such as stiff person syndrome, temporal lobe epilepsy (TLE), limbic encephalitis, cerebellar ataxia (CA), and ocular movement disorders, which are usually chronic and difficult to treat. GAD-related TLE has poor response to anti-seizure medications and immune therapies, and epilepsy surgery is rarely successful. We report on a 47-year-old female with history of migraine, autoimmune thyroid disease, ankylosing spondylitis, and drug-resistant TLE. A video electroencephalography recorded frequent seizures with temporo-insular semiology, correlating to left temporal epileptiform activity and left mesiotemporal hyperintensity on magnetic resonance imaging. GAD autoimmunity was confirmed by very high GAD antibody titers in serum and cerebrospinal fluid. Steroids, immunoglobulins, and cyclophosphamide had no effect, and selective left amygdalectomy was performed based on very restricted hypermetabolism on positron-emission tomography. After transient seizure freedom, significant epilepsy improvement was observed in spite of memory decline. Transient worsening was noted 1 year later during diabetes mellitus manifestation and 5 years later during presentation of progressive CA, which stabilized on rituximab treatment. We believe this case illustrates the diversity and the frequent overlap of GAD-associated disorders, the need of early and aggressive immunotherapy in severe patients, as well as the possible benefit from epilepsy surgery in some GAD-TLE.

## Introduction

Glutamic acid decarboxylase antibodies (GAD-Abs; against the enzyme isoform GAD65) are usually associated with chronic conditions, increasingly recognized during the last three decades. Besides type-1 diabetes mellitus (DM1), GAD-Abs have been associated with a number of neurological syndromes, such as stiff-person syndrome (SPS), cerebellar ataxia (CA), limbic encephalitis (LE) and temporal lobe epilepsy (TLE), ocular movement disorder, and myelitis (1–3). Diverse clinical manifestations are thought to be dictated by GAD-Ab specificity, targeting different epitopes in the catalytic domain of the enzyme (1). Nonetheless, because of massive overlap in epitope recognition, presentations with signs and symptoms of several affected systems may be observed (1, 3, 4). Common features of GAD-Ab-associated neurological disorders are frequent comorbidity with other systemic autoimmune diseases, possibility of developing overlap syndromes, and poor to moderate response to immunotherapies. Here, we present a case with refractory TLE, who was historically diagnosed with migraine, autoimmune thyroid disease, and ankylosing spondylitis (AS). A delayed diagnosis of GAD-Ab autoimmunity was established, and DM1 and CA developed 6 and 10 years after the epilepsy onset, respectively. Immunotherapy with steroids, immunoglobulins, and cyclophosphamide had no effect on seizures, while selective amygdalectomy achieved substantial epilepsy improvement. Rituximab treatment led to stabilization of CA and further seizure reduction.

## Case Presentation

A 42-year-old woman presented for presurgical evaluation due to very frequent epileptic seizures. No febrile convulsions or significant medical antecedents in childhood were reported except for rare migraine attacks since puberty. Historically, the patient was operated at the age of 32 for autoimmune Hashitoxicosis (unfortunately, detailed medical documentation is missing) and was on treatment with 100 μg L-thyroxin. At the age of 36, she was diagnosed with HLA-B27-negative AS based on 6-month clinical manifestation of progressive (predominantly low) back and hip pain, 3-plane limitation of lumbar spine mobility, morning stiffness improving with motion, and confirmation by laboratory (elevated C-reactive protein and negative rheumatoid factor) and radiological findings (bilateral sacroiliac joints changes, syndesmophytosis, and enthesitis in neck and lumbar spine on X-ray). Anti-tumor necrosis factor (anti-TNF) therapy with etanercept (ETN) for 2 years led to AS remission and was discontinued due to tuberculosis (TBC) treated with 4-drug regimen for 4 months.

The epilepsy started at the of age 37 with six bilateral tonic-clonic seizures in sleep. A few months later, focal seizures with preserved or impaired awareness started, initially only few per month but later became weekly and in clusters. According to patient description, they did not change over time and occurred in awake state, and were characterized by goosebumps in the back of the neck, left shoulder, and left arm, followed by a cold sensation in the left hemibody, mostly in the upper extremity and shoulder, subsequent unpleasant, strong and sharp smell, “déjà-vu”/“déjà-vécu” experiences, and trembling of both hands, more on the right. Sometimes, because of a feeling of “breathlessness,” she tried to breathe more deeply and frequently. Her relatives confirmed that usually she could warn at onset. Afterwards, motion arrest, eye closure, oral automatisms and facial flush were observed. Her right hand became stiff and immobile, while the left hand was squeezing. Postictally she could not speak and respond for minutes. Several anti-seizure drugs (ASDs) had no or minimal efficacy (oxcarbazepine and levetiracetam), and some of them had also marked adverse effects (valproate and carbamazepine). Therefore, the therapy consisted of lamotrigine (LTG) and low-dose clonazepam (CZP). Standard electroencephalograms (EEGs) were reported as showing left temporal epileptic focus, and four brain magnetic resonance imaging (MRI) examinations were interpreted as demonstrating left hippocampal sclerosis (HS). Memory problems appeared 2 years after epilepsy onset and increased 5 months before admission, when seizure frequency reached 30 to 40 per day.

**Figure 1:**
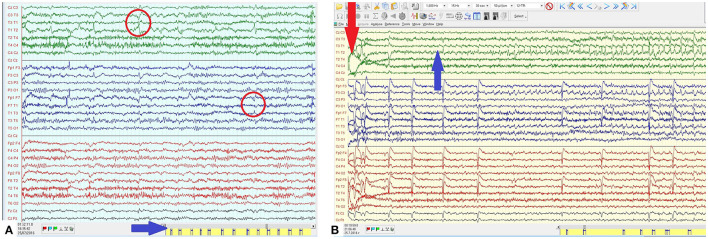
**(A)** EEG in wakefulness with left temporal sharp- and spike-wave (SW) complexes with maximum and phase reversal on T1-T3 (red arrow). Individual seizures occurring at fairly regular interval and high frequency (blue arrow). **(B)** Ictal EEG epoch from the clinical seizure onset (red arrow) demonstrating the evolution of the left temporal ictal discharge consisting of rhythmic SW with an increasing amplitude and a spread mostly to the central region.

During 3-day video-EEG monitoring, more than 150 seizures were recorded. They lasted 40–60 s and correlated to left temporal ictal change of initial attenuation and subsequent rhythmic epileptiform discharge (Figure 1A). In wakefulness, seizures occurred at fairly regular intervals of 3 to 4 per hour (Figure 1B). Rapid titration of topiramate and intravenous (IV) phenytoin did not reduce seizure frequency.

**Figure 2:**
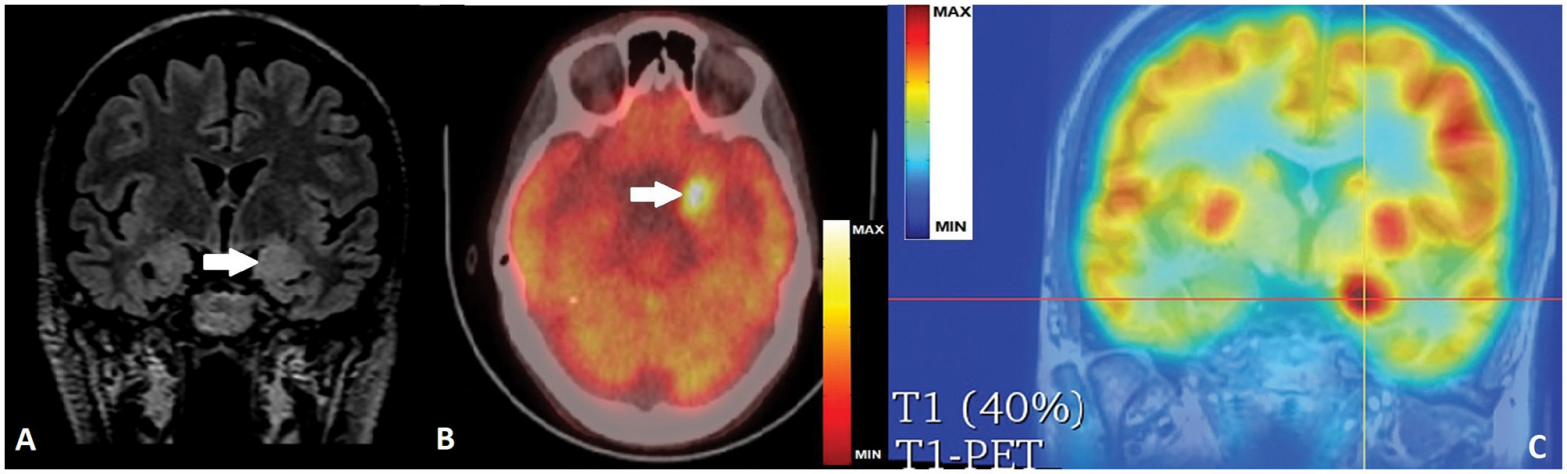
**(A)** Coronal FLAIR MRI with bi-mesiotemporal hyperintensity, more prominent on the left, with increased volume of left amygdala (white arrow). **(B)** Axial PET-CT scan with hypermetabolic left mesiotemporal spot(white arrow). **(C)** PET-T1 MRI co-registration with clear hyperactivity in the left amygdala (crossed lines' center).

Brain MRI demonstrated bilateral mesiotemporal hyperintensity with clear left predominance and enlarged left amygdala (Figure 2A). Neuropsychological testing confirmed verbal memory deficit. Based on clinical history, presentation, and results from the examinations performed, we strongly suspected an autoimmune etiology. Serum and cerebrospinal fluid (CSF) autoimmunity testing by ELISA (Oxford University Hospitals Neuroimmunology Laboratory) was negative for anti-Caspr2, anti-NMDAR, anti-AMPAR-1/2, anti-GABA-b, anti-VGCC, anti-VGKC, and anti-LGI1 Ab, but very high anti-GAD-Ab titers of > 50,000 IU/ml (serum) and >10,000 IU/ml were (CSF) detected. Immunotherapy was immediately started with IV methylprednisolone (MPR), and later continued with IV immunoglobulins (IVIG) and cyclophosphamide, with only brief and transient effect despite decreased serum anti-GAD Ab-titers (15,000 IU/ml).

Control brain MRI did not show progression of mesiotemporal abnormalities but fluorodeoxyglucose-positron emission tomography (FDG-PET) revealed very restricted hypermetabolism in the left amygdala correlating to an ictal event during the examination, as reported by the patient (Figures 2B,C). Because of lacking efficacy of ASDs and immunotherapy, and after thorough discussions with the patient and relatives on the chances and risks of epilepsy surgery, we performed a very selective left amygdalectomy. Postoperatively, the patient was 3 months seizure-free but later continued to experience shorter focal aware/unaware seizures mostly with behavioral arrest, decreased/absent responsiveness, and milder dyscognitive features but without the initial vegetative/somatosensory and the later motor signs. Seizure frequency stabilized at 3 to 4 per month with no change in ASDs, an outcome corresponding to Engel Class IIIA (5).

One year later, the patient was diagnosed with DM1 that was initially difficult to compensate, and transient increase in seizure frequency of up to 1–2 daily for about 2 months was also noted. Later on, with Insulin Apart 6 UI t.i.d. and Insulin Degludec 16 UI q.d., the condition with regard to both DM1 and TLE (weekly non-disabling focal seizures) was stable and did not require further treatment adjustments.

**Figure 3:**
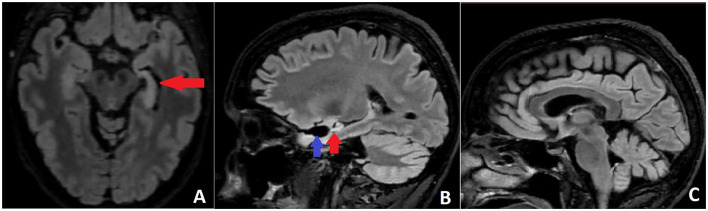
**(A)** Axial FLAIR MRI with left HS (red arrow). **(B)** Sagittal FLAIR MRI scan demonstrating the postoperative defect (blue arrow) and the left HS (red arrow). **(C)** Sagittal FLAIR MRI without obvious signs of cerebellar and brainstem atrophy.

Approximately 4 years after the operation, rare episodes of dizziness and falls were reported and interpreted by the patient as probable seizures. They were not recorded on VEEG, but right temporal epileptiform activity was registered, and lacosamide (LCM) was added. The patient continued to report intermittent unsteady gait, diplopia, vertigo, and more frequent falls, related or not to LCM intake. No changes in the neurological exam, MRI, and EEG were found; therefore, these complaints were attributed to LCM and prompted its replacement with brivaracetam without any improvement. Gradually, over the next 5 months, the patient developed a full-blown picture of CA with severe locomotor ataxia, dysdiadochokinesis, dysmetria, nystagmus, and dysarthria. Brain MRI did not reveal obvious signs of cerebellar atrophy but only progress in left HS (Figure 3). Paraneoplastic auto-Ab testing was negative for anti-Hu (ANNA-1), anti-Ri (ANNA-2), anti-Yo (PCA-1), anti-PNMA2 (Ma2/Ta), anti-Tr (DNER), anti-amphiphysin, anti-CV-2, anti-Sox-1, anti-ZIC4, anti-recoverin, and anti-titin Ab, but high anti-GAD65 titers of 67 U/ml persisted (positive if ≥ 10 U/ml, strong positive ≥ 50 U/ml; [10 U/ml ≈180 IU/ml]).

Immunotherapy with IV MPR and IVIG had no effect; therefore, rituximab treatment was started. After the first application, marked but transient improvement in CA was noted, and after the second dose, the condition stabilized. Further decrease in seizure frequency to two per month was observed as well, but due to the locomotor and distal limb ataxia at present, the 47-year-old woman is independent and able to fulfill her usual activities at home only. In addition, neuropsychological testing before, 6 months after and most recently, 4.5 years after surgery, demonstrated significant worsening of the memory function from mild short-term verbal memory deficit prior to the selective left amygdalectomy to marked verbal and non-verbal memory decline, favoring continuing underlying bilateral damage, regardless of the stable and even improving epilepsy situation with several EEGs free of left and right temporal epileptiform or slow-wave activity.

**Figure 4:**
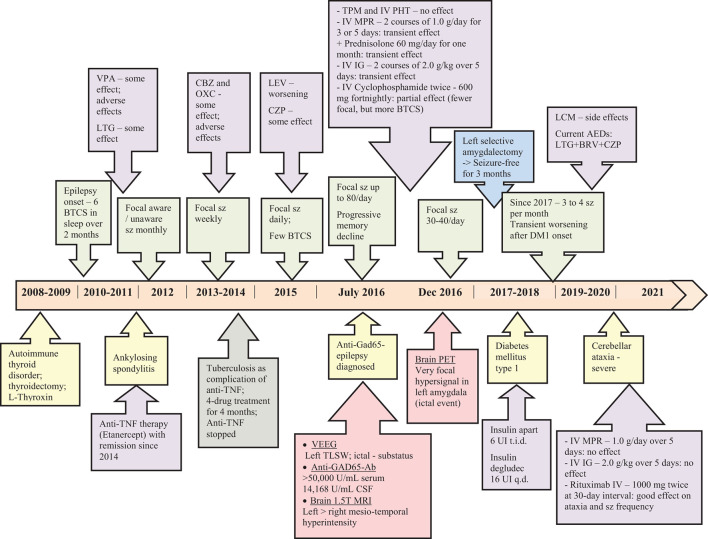
Timeline with diseases' onset and course, the diagnostic work-up, relevant results and therapeutic management.

The timeline of diseases' onset and course, most important diagnostic workup with relevant results, and therapeutic interventions including doses of the medications are presented in Figure 4.

## Discussion

After SPS and CA, GAD-TLE is considered the third most common GAD-associated neurological syndrome and one of the most common types of autoimmune epilepsy (3). GAD-Ab association must be suspected in cases with no obvious cause of TLE, and it has been postulated that GAD-Ab need to be found in high serum titers or to be detected in CSF to prove the causal relationship (1, 2, 4, 6, 7). In our case, a very high GAD-Ab titer was found both in serum and CSF, where the recognized abnormal level is >1,000 IU/ml by ELISA (2), and similar to previous reports, the serum level was much higher than that in CSF (7, 8). Although the serum GAD-Ab titer significantly decreased already after the first IV MPR trial, the disease course did not correlate to this Ab-reduction, since very frequent disabling seizures persisted and later on, two other GAD-related conditions (DM1 and CA) developed as well. In our case, the lacking clinical response to the immunomodulatory treatment despite the positive “relative” trend of Ab-decrease confirms important previous observations that without early immunotherapy this autoimmune disorder has a chronic and hard to treat course (9, 10). Obviously, in such difficult cases, GAD-Ab titer changes seem not be an effective indicator of the ongoing inflammation, and the clinical picture with treatment response is the only guide for short- and long-term therapeutic decisions (10).

As in the other GAD-associated neurological syndromes, comorbidity with other autoimmune disorders, such as DM1, could be present in >1/3 of patients with GAD-TLE/LE (11). It was found that usually the epilepsy preceded DM1 in patients with high GAD-Ab titers (12). Our case supports this observation, as TLE developed 6 years before DM1.

An important issue in our case is the possibility of a causal relationship of the autoimmune neurological disorders to the preceding anti-TNF treatment for AS. It is well known that the fundamental change in the treatment of diverse chronic inflammatory diseases brought by anti-TNF drugs increasingly raises many concerns about the safety of those agents because of various adverse events during this targeted biological treatment (13, 14). Our patient suffered from TBC, the most frequent opportunistic infection, after 2-year treatment with ETN, thus exhibiting one of the most frequent anti-TNF therapy complications, as TNFα is critical for localizing and preventing reactivation of latent mycobacterial TBC infection (14).

As to the autoimmunity, up to now, it has been reported that all anti-TNF drugs induce the development of anti-drug Ab, and that the neurological complications related to those agents are demyelinating central and peripheral nervous system diseases (13, 14). Several speculative hypotheses have been proposed to explain the mechanism of the possible relationship between anti-TNF and demyelination, but none is considered unique and adequate; moreover, several factors argue against the true association between anti-TNF therapy and demyelinating disease (14). Based on t available literature data and uncertainties on this issue, we do not consider the GAD-Ab-associated neurological disorders in our case to be related to the anti-TNF treatment, but rather we adopt the view that they are part of a multisystem autoimmune syndrome, especially having in mind the initial manifestation of a (poorly documented) autoimmune thyroid disorder, i.e., Graves' disease alone or in combination with Hashimoto's thyroiditis, as already well-documented (15).

Glutamic acid decarboxylase antibody (GAD-Ab)-associated focal epilepsy seems not to be restricted to the TL and hippocampus; the limbic system is most often affected bilaterally, and insular hypometabolism on PET could serve as an important diagnostic clue (10, 16). In our case, the seizure semiology was in favor of early insular involvement, as ictal onset was with autonomic and somatosensory signs, suggesting generation in the insula or temporo-insular circuit (10, 16–18). Autoimmune etiology in seizures with piloerection has been reported in autoimmune TLE (19–23), and to our knowledge there is only one published case of such semiology in GAD-associated temporo-perisylvian epilepsy (23).

Our patient presented for presurgical evaluation 5 years after epilepsy onset with dramatic seizure frequency increase over a few months, concomitantly to progressive memory disturbances, bilaterally hyperintense and swollen mesio-temporal structures, and high GAD-Ab levels. Although one major diagnostic criterion for definite limbic encephalitis is the subacute onset (<3 months) of working memory deficits and seizures (24), we believe that the other findings in our case (bilateral FLAIR-T2 mesio-temporal abnormalities, EEG showing epileptiform and slow-wave activity involving the temporal lobes, and high GAD Ab-titers both in serum and CSF) are sufficient to reasonably exclude alternative causes (24), and to speculate that the condition of our patient could be regarded as an exacerbation of “chronic” LE rather than just worsening of chronic TLE.

In the above cornerstone position article (24), it has been underlined that brain FDG-PET is much more often abnormal than time-matched MRI in cases of autoimmune LE (24–26). Moreover, hypermetabolic PET findings were found to be related to the specific antibody-type, i.e., all patients with Ab against intracellular antigens (such as GAD) showed mesio-temporal PET hyperactivity more frequently than patients with Ab against surface antigens (25). Our patient confirmed that during the PET examination she has had a usual seizure. Therefore, we considered the circumscribed hyperintense area corresponding to the left amygdala as presumable hypermetabolic ictal onset zone, most probably on top of a very active inflammation in the mesiotemporal complex, rather than as possible hypometabolism on the right side.

The surgical outcome in GAD-TLE is worse than in other refractory TLE, as it has been demonstrated by several studies that the majority of operated cases had minimal or no improvement, and that only few became seizure-free (9, 12, 27–29). Moreover, even when selective amygdalohippocampectomy controlled or improved the seizures, in combination with immunotherapy, the long-term performance in verbal and figural memory was worse than with immunomodulation only (27). It is suggested that high seizure recurrence and memory decline over the long run are related to the frequent bilaterality, widespread involvement of the limbic system by GAD-autoimmunity, and structural changes in the third stage of GAD-TLE rather than to the ongoing inflammatory process (2, 16). In our case, despite significant postoperative seizure decrease, HS on MRI and memory problems progressed; therefore, continuing autoimmune activity seems to be of major role, since DM1 and CA manifested as well.

Cerebellar ataxia (CA) is the second most frequent GAD-autoimmune disorder and is often comorbid with DM1 (3, 30–32). Although infrequent, association with epilepsy is possible, including refractory GAD-TLE preceding the CA by up to 15 years (32). Similar to what was already described (32), the full-blown CA in our case was antedated by intermittent episodes of diplopia, vertigo, and ataxia wrongly attributed to new AED. Most likely, the transient episodes represented subtle dysfunctions that in already proven GAD autoimmunity should raise concern about impending CA and prompt evaluation and immunotherapy to increase the chance of improvement (32).

Patients with delayed diagnosis of GAD-associated epilepsy were found to be usually ASD-resistant and not responding to immunotherapy (29). In such difficult cases, “the interplay between AEDs and immunotherapy” (29) is the cornerstone of management, which, in our patient, was further complicated by the subsequent manifestation of DM1 and CA. The treatment approach in every case must be individually oriented, and in this regard, epilepsy surgery cannot be excluded as an option for improvement. We hope that our case could be accepted as an example of complex clinical scenarios, in which tailored and very selective epilepsy surgery might be a useful treatment option to reduce seizure frequency and improve quality of life.

## Data Availability Statement

The original contributions presented in the study are included in the article/supplementary material, further inquiries can be directed to the corresponding author/s.

## Ethics Statement

All clinical data in this case report were provided by the patient or collected by the authors with the consent of the patient. Written informed consent was obtained from the patient for the publication of this report.

## Author Contributions

PD participated in patient management, clinical data analysis, and writing of the article. KM participated in patient management and revision of the article. All authors contributed to the article and approved the submitted version.

## Conflict of Interest

The authors declare that the research was conducted in the absence of any commercial or financial relationships that could be construed as a potential conflict of interest.

## Publisher's Note

All claims expressed in this article are solely those of the authors and do not necessarily represent those of their affiliated organizations, or those of the publisher, the editors and the reviewers. Any product that may be evaluated in this article, or claim that may be made by its manufacturer, is not guaranteed or endorsed by the publisher.
